# Three Cases of Neurologic Syndrome Caused by Donor-Derived Microsporidiosis 

**DOI:** 10.3201/eid2303.161580

**Published:** 2017-03

**Authors:** Rachel M. Smith, Atis Muehlenbachs, Joanna Schaenmann, Sanjiv Baxi, Sophia Koo, Dianna Blau, Peter Chin-Hong, Anna R. Thorner, Matthew J. Kuehnert, Kristina Wheeler, Alexis Liakos, Jonathan W. Jackson, Theresa Benedict, Alexandre J. da Silva, Jana M. Ritter, Dominique Rollin, Maureen Metcalfe, Cynthia S. Goldsmith, Govinda S. Visvesvara, Sridhar V. Basavaraju, Sherif Zaki

**Affiliations:** Centers for Disease Control and Prevention, Atlanta, Georgia, USA (R.M. Smith, A. Muehlenbachs, D. Blau, M.J. Kuehnert, J.W. Jackson, T. Benedict, A.J. da Silva, J.M. Ritter, D. Rollin, M. Metcalfe, C.S. Goldsmith, G.S. Visvesvara, S.V. Basavaraju, S. Zaki);; University of California, Los Angeles, California, USA (J. Schaenmann);; University of California, San Francisco, California, USA (S. Baxi, P. Chin-Hong);; Brigham and Women’s Hospital, Boston, Massachusetts, USA (S. Koo, A.R. Thorner, A. Liakos);; Harvard Medical School, Boston (S. Koo, A.R. Thorner, A. Liakos);; OneLegacy, Los Angeles (K. Wheeler)

**Keywords:** organ transplants, microsporidia, microsporidiosis, communicable diseases, neurologic syndrome, meningitis/encephalitis, fungi

## Abstract

In April 2014, a kidney transplant recipient in the United States experienced headache, diplopia, and confusion, followed by neurologic decline and death. An investigation to evaluate the possibility of donor-derived infection determined that 3 patients had received 4 organs (kidney, liver, heart/kidney) from the same donor. The liver recipient experienced tremor and gait instability; the heart/kidney and contralateral kidney recipients were hospitalized with encephalitis. None experienced gastrointestinal symptoms. *Encephalitozoon cuniculi* was detected by tissue PCR in the central nervous system of the deceased kidney recipient and in renal allograft tissue from both kidney recipients. Urine PCR was positive for *E. cuniculi* in the 2 surviving recipients. Donor serum was positive for *E. cuniculi* antibodies. *E. cuniculi* was transmitted to 3 recipients from 1 donor. This rare presentation of disseminated disease resulted in diagnostic delays. Clinicians should consider donor-derived microsporidial infection in organ recipients with unexplained encephalitis, even when gastrointestinal manifestations are absent.

## Abstract 2

*Encephalitozoon cuniculi* was transmitted from an infected donor to 3 solid organ recipients, 1 of whom died.

Each year in the United States, ≈30,000 solid organ transplants are performed (*1*). It is estimated that 0.3%–2.0% of transplants may be complicated by donor-derived infection, most commonly of bacterial or viral origin (*2*–*4*). Parasitic and fungal infections, including microsporidiosis, make up a minority of donor-derived infections (*2*–*4*). Maintaining a high index of suspicion for donor-derived infection in solid organ transplant recipients and prompt investigation of illness suspected to be donor derived are critical because multiple recipients often receive solid organs from a common donor. Thus, identification of donor-derived infection in 1 recipient has consequences for the clinical care of the other recipients.

Potential donor-derived disease transmission events are reported to the Organ Procurement and Transplantation Network (https://optn.transplant.hrsa.gov/) per policy and reviewed by the Network’s ad hoc Disease Transmission Advisory Committee, which categorizes each by the likelihood of disease transmission. Through representation on this advisory committee, the Centers for Disease Control and Prevention (CDC), with support from state and local health departments, leads investigations of select cases of public health importance. In April 2014, CDC was notified of a renal transplant recipient hospitalized with signs and symptoms of encephalitis (Figure 1). Postmortem testing revealed infection with microsporidia, and concern was raised for donor-derived central nervous system (CNS) infection. We conducted an investigation to 1) identify other ill recipients from the common donor, 2) determine whether the illness was donor derived, and 3) make treatment recommendations for the surviving recipients.

**Figure 1:**
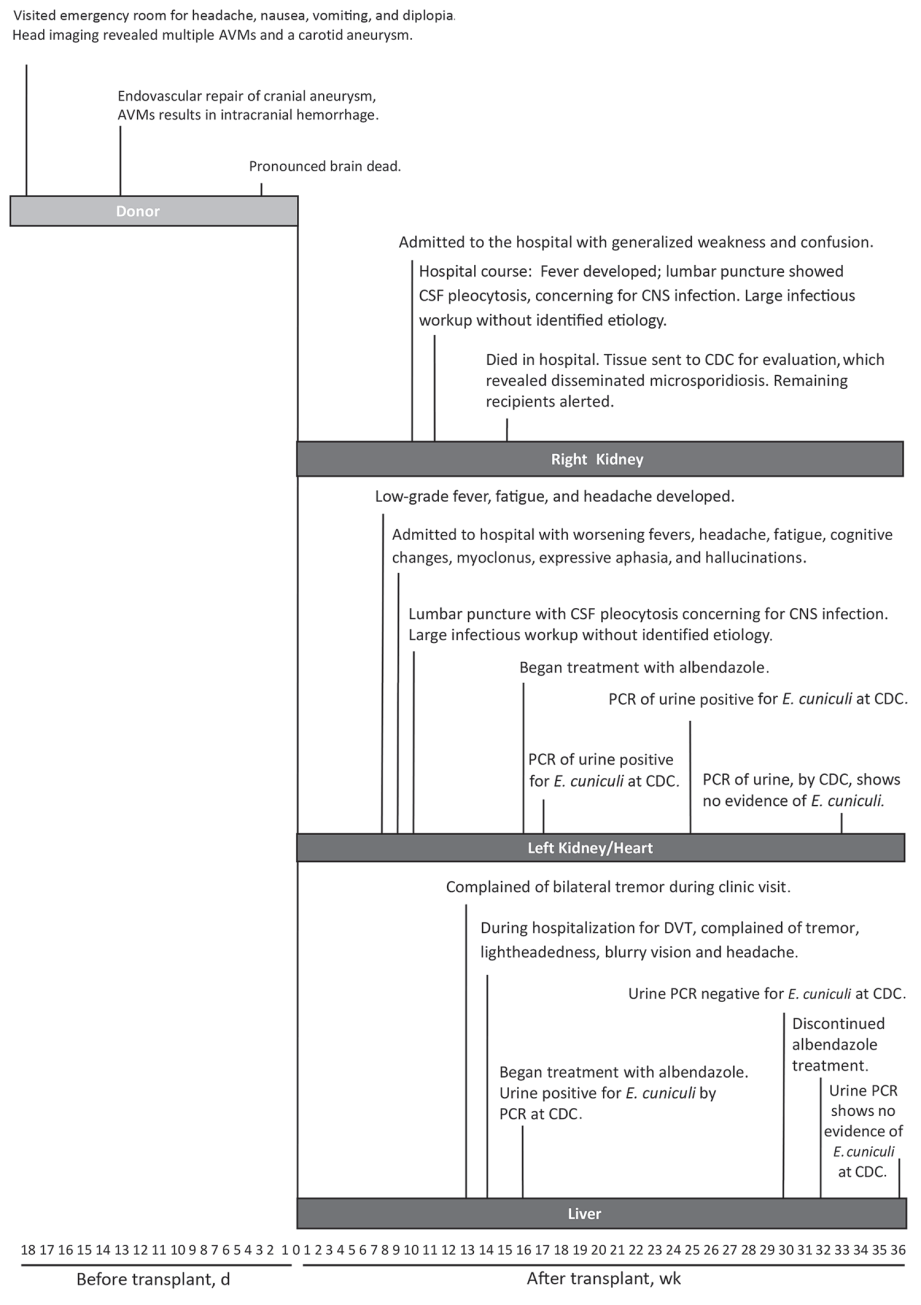
Timeline of events for transplant donor and 3 solid organ recipients with microsporidiosis (*Encephalitozoon cuniculi*). AVM, arteriovenous malformation; CDC, Centers for Disease Control and Prevention; CNS, central nervous system; CSF, cerebrospinal fluid; DVT, deep vein thrombosis.

## Methods

Medical records from the organ donor and all recipients were reviewed to describe clinical course, diagnostic testing, and event timelines. Epidemiologic investigation identified other ill recipients. The donor’s next of kin were interviewed to ascertain potential risk factors for infectious diseases, including microsporidiosis. Because this investigation was considered a public health emergency, it was not subject to institutional review board approval. 

A serum sample from the organ donor and available specimens from all recipients were sent to CDC for further testing to identify an infectious etiology. These tests included histopathology, in vitro culture immunohistochemistry (IHC), PCR, and transmission electron microscopy. Serum immunofluorescence antibody testing (IFA) was performed on available serum samples.

### Cell Culture and Serum Testing

We inoculated recipient urine specimens onto monkey kidney cells (Vero E6) and human embryonic lung fibroblasts by using established methods (*5*). Cultures were treated with 2% sodium dodecyl sulfate and passaged to eliminate cytomegalovirus. For IFA, *Encephalitozoon cuniculi* reference strain cultures were suspended in phosphate-buffered saline (PBS) at a concentration of 10^8^ spores/mL. We added 10 μL of this suspension to each well (10^6^ spores/well), allowed the slides to air dry, and stored them at −80°C before use.

Serum samples were diluted 1:2 in 25 μL PBS along with previously established positive (titer 1:4,096) and negative (titer 1:32) control serum obtained from the CDC free-living amebic infections laboratory. The serum samples were then diluted to 1:4,096 by 2-fold dilution. We added 10 μL from each dilution to the wells of the previously made *E. cuniculi* IFA slides, which were then incubated at 37°C for 30 min. The slides were washed 3 times in PBS before the addition of a 1:100 dilution of fluorescein isothiocyanate–conjugated goat anti-human IgG (Cappel Laboratories, Cochranville, PA, USA) in PBS with 3 μL/mL Evans Blue (Fisher Scientific, Pittsburgh, PA, USA) counterstain. After another 30-min incubation at 37°C, the slides were again washed 3 times in PBS, dried, and mounted with glycerol mounting media. Serum titers were determined by observing the slides under a fluorescence microscope. Antibody titers were considered positive at cutoffs of >1:16 for immunocompromised and >1:64 for immunocompetent persons (*6*).

### Histopathology, Immunohistochemistry, and Transmission Electron Microscopy

We processed formalin-fixed paraffin-embedded tissues by using standard histologic methods. IHC was performed by using a polymer-based indirect immunoalkaline phosphatase detection system with a fast red chromogen kit (Biocare Medical, Concord, CA, USA). The microsporidia IHC assay used rabbit anti–*E. cuniculi* serum at a 1:1,000 dilution as previously described (*7*). In addition, IHC assays for lymphocytic choriomeningitis virus, measles virus, and *Trypanosoma cruzi* were performed as previously described (*8*–*10*).

For transmission electron microscopic examination, we evaluated a paraffin-embedded section by on-slide embedding as previously described (*11*). The urine cell pellet and microsporidia culture isolates were transferred to buffered 2.5% glutaraldehyde and 1% osmium tetroxide, embedded in a mixture of Epon substitute and Araldite (Ted Pella, Inc., Redding, CA, USA), sectioned, and stained with uranyl acetate and lead citrate.

### Molecular Techniques

DNA was extracted from unpreserved clinical samples by using a DNeasy Blood & Tissue DNA extraction kit (QIAGEN, Valencia, CA, USA). DNA was extracted from the formalin-fixed paraffin-embedded tissues by using a QIAamp DNA Mini Kit (QIAGEN) as previously described (*7*,*12*). PCR primers (concentration 15 μmol/L each) specific for *E. cuniculi* small subunit ribosomal RNA gene were used (*7*,*12*). We performed PCR by using AmpliTaq Gold PCR Master Mix (ThermoFisher Scientific, Grand Island, NY, USA) and 5 μmol of each primer at an annealing temperature of 65°C. All DNA extracts were subjected to PCR. Positive and negative controls were included in every PCR run. Any specimen that resulted in a 551-bp fragment was considered positive for the presence of *E. cuniculi* DNA.

## Results

### Right Kidney Recipient

The right kidney recipient was a man with history of end-stage renal disease resulting from type 2 diabetes mellitus. He received basiliximab to induce immunosuppression, and at discharge he received tacrolimus, mycophenolate mofetil, and prednisone to maintain immunosuppression. His immediate postoperative course was complicated by delayed graft function and anemia. Approximately 10 weeks after transplantation, generalized weakness and confusion developed, and he was admitted to the hospital for evaluation. Initially his clinical condition was thought to result from noninfectious causes, such as medication side effects. However, during his hospitalization, fever, pancytopenia, and acute renal failure developed, and his mental status worsened. Lumbar puncture was performed, and cerebrospinal fluid analysis revealed 10 leukocytes/μL, which led to concern for viral encephalitis. An extensive evaluation for CNS infection was initiated, and test results were negative for bacterial, fungal, viral, and parasitic causes (Table 1). Despite empirically prescribed broad-spectrum antimicrobial drugs, the recipient’s illness progressed, his obtundation worsened, and hypotension required vasopressors. He died ≈15 weeks after transplantation. An autopsy was performed at the local hospital, and tissues were submitted to CDC for examination. Four days after the recipient’s death, examination at CDC of the deceased recipient’s renal allograft demonstrated intracellular organisms consistent with microsporidia. Clinicians caring for the other recipients were immediately notified.

**Table 1:** Infectious disease testing performed for 3 transplant recipients with donor-derived microsporidiosis

Pathogen	All recipients	Right kidney recipient	Left kidney/heart recipient	Liver recipient
Bacterial	Bacterial culture (blood, urine)	*Mycoplasma*	*Borrelia burgdorferi*	*Treponema pallidum*
		*Mycobacterium tuberculosis*	*Anaplasma/Ehrlichia*	
		*Borrelia burgdorferi*	*Treponema pallidum*	
		*Brucella* spp.	*Tropheryma whipplei*	
		*Rickettsia* spp.		
		*Pneumocystis jiroveci*		
		*Legionella* spp.		
Viral	Cytomegalovirus	Enterovirus	Enterovirus	None
	Herpes simplex virus	Lymphocytic choriomeningitis	Adenovirus	
	Epstein-Barr virus	Measles virus	Lymphocytic choriomeningitis	
	Parvovirus	JC virus	Measles virus	
	HIV	Human herpesvirus-6	JC virus	
		Viral fecal cultures	Human herpesvirus-6	
		BK virus		
		Human T-cell lymphotropic virus		
		Meningoencephalitis panel*		
Fungal/parasitic/other	*Cryptococcus* spp.	*Coccidioides* spp.	(1→3)β-D-glucan	*Toxoplasma gondii*
		*Aspergillus* spp.	*Galactomannan*	
		*Strongyloides* spp.	*Coccidioides* spp.	
			*Cryptococcus* spp.	
		*Schistosoma* spp.	*Toxoplasma gondii*	
		*Babesia* spp.	*Babesia* spp.	
			14–3-3 testing for prion disease (cerebrospinal fluid)	

*Panel contains the following viruses: West Nile, Lacrosse, Eastern equine encephalitis, St. Louis encephalitis, Western equine encephalitis, lymphocytic choriomeningitis, herpex simplex 1 and 2, adenovirus, influenza A and B, measles, mumps, varicella, coxsackie A and B, echovirus, cytomegalovirus.

**Figure 2:**
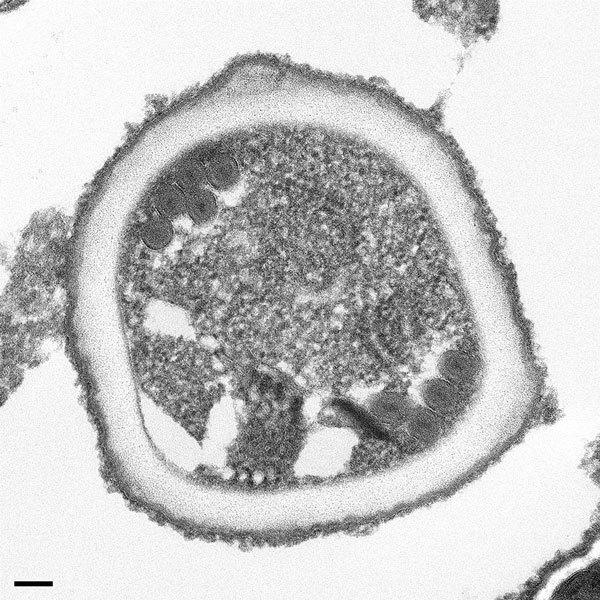
Transmission electron microscopy of microsporidia identified in allograft samples from right kidney recipient. The organism shows cross-sections through the polar tube with up to 6 coils and a unikaryotic nucleus, which is characteristic of *Encephalitozoon cuniculi*. Scale bar indicates 100 nm.

Subsequent PCR of DNA extracts from the right renal allograft revealed the species to be *E. cuniculi*, and electron microscopy showed a polar tubule arrangement characteristic for *E. cuniculi* (Figure 2). The recipient’s CNS tissue also was positive for *E. cuniculi* by histopathology and PCR, which showed microsporidia associated with glial nodules and the leptomeninges, the latter with perivascular inflammation. No arteritis or aneurysmal change in the vessels of the CNS were observed. Immunohistochemistry of CNS tissue also provided positive results (Table 2). IHC results were negative for lymphocytic choriomeningitis virus, measles virus, and *T. cruzi*.

**Table 2:** Transplant recipient testing for microsporidiosis, by specimen and test type*

Patient	Specimen	IHC	TEM	PCR†	Culture
Right kidney recipient	Renal allograft	+	+	+	Not performed
	CNS tissue‡	+	+	Not performed	Not performed
Left kidney/heart recipient	Renal allograft	–	Not performed	+	Not performed
	Cardiac allograft	Indeterminate	-	–	Not performed
	Bone marrow	–	Not performed	–	Not performed
	Urine	Not performed	Not performed	+	Not performed
Liver recipient	Urine	+	+	+	+

*CNS, central nervous system; IHC, immunohistochemistry; TEM, transmission electron microscopy; +, positive; –, negative. †All PCR positive tests confirmed *Encephalitozoon cuniculi* ‡CNS tissue included cortex and brainstem.

### Left Kidney and Heart Recipient

The recipient of the left kidney and heart was a woman in whom coronary vasculopathy and calcineurin inhibitor–induced nephropathy had developed after a cardiac transplant 21 years earlier. To induce immunosuppression before the upcoming kidney and heart transplant, she received a 5-day course of antithymocyte globulin; to maintain immunosuppression, she received tacrolimus, mycophenolate mofetil, and prednisone. Her immediate posttransplant course was complicated by delayed renal graft function, as well as delirium and tremors thought to be associated with uremia and excessive levels of tacrolimus. The delirium and tremors resolved with improved graft function, and her immunosuppression regimen was changed to include cyclosporine rather than tacrolimus. Two months after transplantation, she experienced low-grade fever, fatigue, and headache. Testing for infectious causes, including herpesviruses, enterovirus, West Nile virus, *Borrelia burgdorferi*, and *Cryptococcus*, were negative. Mycophenolate mofetil, valganciclovir, and trimethoprim/sulfamethoxazole prophylaxis were temporarily discontinued because of leukopenia. A week later, she experienced continuing fevers up to 102°F; worsening frontal headache; fatigue; pain in her shins, wrists, and elbows; cognitive slowing; prominent myoclonus; expressive aphasia; and visual hallucinations. She was markedly leukopenic; absolute neutrophil count was 430 cells/μL. Brain magnetic resonance imaging did not reveal mass lesions, infarct, or hydrocephalus. Cerebrospinal fluid testing revealed 17 leukocytes/μL and a protein level of 80.4 mg/dL; however, results of extensive testing for an infectious etiology were negative (Table 1). Routine endomyocardial biopsy samples showed no evidence of rejection; a renal allograft biopsy sample showed evidence of Banff type IIa acute cellular rejection, for which she received 3 doses of methylprednisolone.

After CDC communicated the finding of microsporidiosis in the deceased recipient’s allograft, this recipient empirically received treatment with albendazole at 400 mg twice daily. *E. cuniculi* was detected by PCR from urine obtained at the time of albendazole initiation. Her neurologic symptoms resolved, and after 4 months of albendazole therapy, PCR of urine for *E. cuniculi* was negative. Therapy with albendazole was continued for 1 year. As of July 2016, she remained well without any symptoms of microsporidial infection 1 year after stopping albendazole therapy.

### Liver Recipient

**Figure 3:**
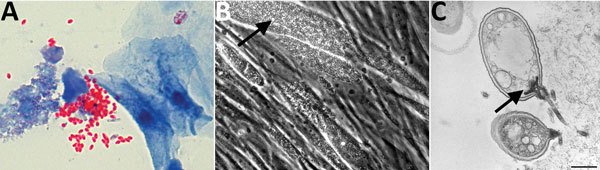
Microsporidia identified in urine samples from liver recipient. A) Urine trichrome stain. Original magnification ×100. B) Cell culture showing microsporidium (arrow). Original magnification ×200. C) Transmission electron microscopic image of infected cell culture with germinating microsporidial spore (arrow). Scale bar indicates 500 nm.

The liver recipient was a man with hepatitis C–associated cirrhosis and hepatocellular carcinoma. At the time of transplantation, he received intravenous methylprednisolone to induce immunosuppression; at the time of discharge, he received tacrolimus, prednisone, and mycophenolate mofetil to maintain immunosuppression. Thirteen weeks after transplant, the patient visited an outpatient clinic and reported bilateral upper extremity tremor, which was thought to result from elevated tacrolimus levels. A week later, the patient was readmitted to the hospital after an outpatient visit for right lower extremity swelling and shortness of breath; the ultimate diagnosis was deep vein thrombosis and pulmonary embolism. During this hospitalization, the recipient reported lightheadedness, headache, blurry vision, and continued tremor. Because of his neurologic symptoms and identification of microsporidiosis in the deceased right kidney recipient, this patient was also empirically given albendazole. The recipient declined lumbar puncture. Subsequently, microsporidia were identified in a urine specimen by trichrome staining performed at the patient’s local hospital (Figure 3, panel A). PCR of the urine specimen confirmed infection with *E. cuniculi*. Microsporidia were also isolated from the urine specimen at CDC after 2 months of cell culture (Figure 3, panel B), and identification was confirmed by IHC and transmission electron microscopy (Figure 3, panel C). The patient’s neurologic symptoms resolved, and subsequent urine PCR for *E. cuniculi* was negative. Because of financial constraints, albendazole was discontinued after 4 months of therapy. Repeated PCRs of urine over the next year remained negative for microsporidia. As of December 2015 (14 months after stopping albendazole), the patient remained well without symptoms of microsporidial infection. 

### Organ Donor

The donor was a middle-aged woman, originally from Mexico but a resident of the United States for several decades. In December 2013, she experienced recurrent and persistent headache, which she attributed to a known history of migraine. The headaches persisted and progressed over several weeks to include nausea, pulsatile tinnitus, and diplopia. Approximately 4 weeks later, she sought care at an emergency department, where brain computed tomography and magnetic resonance imaging revealed several arteriovenous malformations and a right internal carotid saccular aneurysm. She underwent endovascular repair with stenting and coil embolization. This repair was complicated by several episodes of intracranial hemorrhage requiring craniotomy. Despite surgical intervention, her neurologic function continued to decline, and she was eventually declared brain dead. No other symptoms were reported before organ donation. Review of her medical record revealed no reports of gastrointestinal illness during her hospitalization. Interviews with next of kin revealed no clear risk factors for microsporidiosis (e.g., exposure to potentially contaminated water, travel to areas with potentially contaminated drinking water), no gastrointestinal illness before her death, and no travel outside the United States within the previous 12 months.

Because no autopsy was performed on the donor, the only donor specimen available for retrospective testing was archived serum. This sample was tested by microsporidia IFA; a positive titer of 1:2,048 suggested active infection (*13*,*14*).

## Discussion

Microsporidia are a diverse group of intracellular, spore-forming organisms; their molecular taxonomic classification is fungi. Microsporidiosis historically occurred in patients with advanced HIV infection before the era of antiretroviral therapy, and it has increasingly occurred in solid organ and hematopoietic stem cell transplant recipients (*15*–*18*). We describe a cluster of 3 solid organ transplant recipients in whom disseminated microsporidiosis developed, the organism having been transmitted by 1 infected donor. Although a laboratory-confirmed donor-derived cluster of microsporidiosis has been reported (*7*), the cluster reported here is unique in that all 3 recipients experienced neurologic disease in the absence of gastrointestinal signs and symptoms, an extremely rare presentation of disseminated microsporidiosis. Previously, CDC has described infection with West Nile virus, rabies, lymphocytic choriomeningitis virus, and *Balamuthia mandrillaris*, transmitted through solid organ transplantation and manifested as encephalitis among recipients (*19*–*22*). The cluster reported here adds microsporidiosis to the list and points to the need for clinicians to maintain awareness of this pathogen when evaluating transplant recipients who exhibit signs or symptoms suggestive of encephalitis.

The most common presentation of microsporidiosis is gastrointestinal disease causing diarrhea and malabsorption; disseminated disease often involves the urinary tract. *E. cuniculi* has been found in the CNS during postmortem examination of patients who had widely disseminated disease, often without clear preceding neurologic manifestations (*23*–*26*). However, presentation with isolated severe neurologic disease, as seen in the transplant recipients in this cluster, is very rare, although it has occurred in immunocompetent persons (*27*,*28*). This rare presentation probably led to the diagnostic and treatment delays for the patients in this cluster. Nonspecific symptoms, such as weakness and confusion, as seen early in the deceased right kidney recipient in this cluster, may also be difficult to ascribe to an infectious or donor-derived etiology, especially for older recipients with multiple concurrent conditions.

Diagnosis of microsporidiosis is challenging; the organisms are not easily visible with routine Gram staining and do not grow in standard culture media. However, if clinical suspicion exists, rapid diagnosis can be accomplished at clinical laboratories by light microscopy of specimens (usually urine or feces) and use of a modified trichrome stain (Figure 3, panel A). Therefore, diagnosis is dependent on clinicians maintaining a high level of suspicion and obtaining the appropriate local testing or referring samples to specialized institutions that can perform advanced diagnostics, such as PCR or cell culture (Figure 3, panel B). 

In the cluster we report, an extensive evaluation to determine the etiology of illness was performed for the 2 recipients hospitalized with encephalitis. Microsporidiosis was not considered during the 6-week disease course until an autopsy had been performed on the deceased recipient and subsequent specialized studies at CDC revealed the diagnosis. Clinicians should be aware that the clinical presentation of disseminated microsporidiosis can be a neurologic syndrome in the absence of gastrointestinal symptoms. Further research is needed to understand whether a neurotropic variant of *E. cuniculi* might be the cause of isolated severe neurologic microsporidiosis, as occurred in these 3 patients. Furthermore, given that animal studies have shown that encephalitozoonosis can cause vasculitic manifestations, including aortitis (*29*) and artertitis (*30*) in primates, the intracerebral pathology of the donor might have been associated with occult disseminated *E. cuniculi* infection.

Although microsporidial infections have been described for transplant patients, establishing a donor-derived etiology is challenging for many reasons. Microsporidia are transmitted by the fecal–oral route; thus, any exposure to feces, either directly or via contaminated water or soil, probably represents a mode of acquisition. However, little is known about additional risk factors or other ways of acquiring microsoporidial infection. Similarly, little data are available on incubation period, duration of illness/shedding, and likelihood of dissemination in immunocompetent or immunocompromised patients. Therefore, it may not be possible to determine the source of infection in transplant recipients (e.g., derived from transplant vs. acquired from the environment) and the likelihood of underlying donor infection on the basis of clinical course or characteristics alone. However, when a similar infectious syndrome develops in >2 recipients of organs from a common donor, the chance that it is donor-derived increases substantially. After donor-derived infection is suspected, assessing whether a donor had active disseminated or even clinically significant microsporidial infection is also challenging. Although organ procurement organizations are required to keep donor serum samples for postmortem testing, there is no commercially available serologic test for microsporidiosis. IFA testing, along with advanced testing to compare species, as was used in this investigation, require reference laboratory capacity. Although donor disease can be identified through examination of tissues, autopsies are frequently not conducted, and archived tissues are often unavailable. Identification of new and emerging donor-derived infections could be greatly facilitated if donor autopsy rates are increased.

Microsporidia species identification has treatment implications. Although Encephalitozoonidae are susceptible to albendazole, *Enterocytozoon bieneusi*, another common species of microsporidia affecting humans, is not. Although immunocompetent persons may require no treatment or short-course therapy (*31*,*32*), the appropriate treatment duration for immunocompromised patients remains unclear. For patients with HIV/AIDS, immune reconstitution is a key component to treatment. However, in transplant recipients for whom de-escalation of immunosuppression may not be immediately possible, the optimal length of treatment has not been well studied. Although some recommendations suggest 2–4 weeks of therapy, the patients described in this cluster received treatment until demonstration of clearance of infection (as identified through urine PCR). Unfortunately, despite having recently received a resource-intensive intervention such as liver transplant, the expense associated with albendazole (reportedly ≈$2,000/month), a drug that has been available in generic form since the 1980s and is on the World Health Organization Essential Medicine List (*33*), precluded continuing therapy for the liver recipient. High costs of generic drugs with niche markets have been linked to drug shortages, supply disruptions, or, in the case of generic albendazole, consolidations in the generic drug industry, leading to a single manufacturer with the ability to set prices without competition (*34*).

The findings of this investigation are subject to limitations. Because no donor tissue was available for testing, we were unable to differentiate whether the donor’s neurologic complaints and radiographic findings were caused by microsporidial disease or whether she had subclinical disseminated infection. Additionally, the IFA result interpretation has not been standardized for the serologic diagnosis of microsporidiosis. Last, sequencing of *E. cuniculi* strains (e.g., the internal transcribed spacer region of rRNA or whole-genome sequencing) was not performed as part of the public health investigation, and we are thus unable to further investigate strain relatedness. However, we were able to confirm infection with the same species of microsporidia in all 3 recipients and the donor, which is highly suggestive of donor-derived transmission.

Identifying donor-derived disease transmission events and ensuring appropriate treatment and management of recipients requires extensive collaboration. This investigation, as did other donor-derived disease transmissions described by CDC, required close cooperation among clinicians, laboratory scientists, the organ procurement organization, and public health agencies. Clinicians should maintain a high index of suspicion for donor-derived infections and should report any suspected or potential events to the Organ Procurement and Transplantation Network.
